# Cross-Modal Tinnitus Remediation: A Tentative Theoretical Framework

**DOI:** 10.3390/brainsci14010095

**Published:** 2024-01-19

**Authors:** Antoine J. Shahin, Mariel G. Gonzales, Andrew Dimitrijevic

**Affiliations:** 1 Department of Cognitive and Information Sciences, University of California, Merced, CA 95343, USA; mgonzales23@ucmerced.edu; 2 Health Science Research Institute, University of California, Merced, CA 95343, USA; 3 Sunnybrook Research Institute, University of Toronto, Toronto, ON M4N 3M5, Canada; andrew.dimitrijevic@sunnybrook.ca

**Keywords:** audiovisual processing, auditory evoked potentials, cross-modal encoding, EEG, neural oscillations, tinnitus

## Abstract

Tinnitus is a prevalent hearing-loss deficit manifested as a phantom (internally generated by the brain) sound that is heard as a high-frequency tone in the majority of afflicted persons. Chronic tinnitus is debilitating, leading to distress, sleep deprivation, anxiety, and even suicidal thoughts. It has been theorized that, in the majority of afflicted persons, tinnitus can be attributed to the loss of high-frequency input from the cochlea to the auditory cortex, known as deafferentation. Deafferentation due to hearing loss develops with aging, which progressively causes tonotopic regions coding for the lost high-frequency coding to synchronize, leading to a phantom high-frequency sound sensation. Approaches to tinnitus remediation that demonstrated promise include inhibitory drugs, the use of tinnitus-specific frequency notching to increase lateral inhibition to the deafferented neurons, and multisensory approaches (auditory–motor and audiovisual) that work by coupling multisensory stimulation to the deafferented neural populations. The goal of this review is to put forward a theoretical framework of a multisensory approach to remedy tinnitus. Our theoretical framework posits that due to vision’s modulatory (inhibitory, excitatory) influence on the auditory pathway, a prolonged engagement in audiovisual activity, especially during daily discourse, as opposed to auditory-only activity/discourse, can progressively reorganize deafferented neural populations, resulting in the reduced synchrony of the deafferented neurons and a reduction in tinnitus severity over time.

## 1. Introduction

Tinnitus is a phantom sound sensation, often heard as a high-frequency tone, that is experienced by over 50 million people in the United States alone (Source: American Tinnitus Association and National Institute of Health). Chronic tinnitus can be debilitating, causing distress, sleep deprivation, anxiety, and even suicidal thoughts [1,2]. Hence, tinnitus represents a high-priority area in hearing health. The etiology of the disease, the type of sensation, and possible causes and remedies vary substantially [3,4,5]. Tinnitus heterogeneity is reflected across several dimensions [6], which include perception of the type of sound (e.g., hissing, pitched tone), its form of manifestation (e.g., occasional, chronic), its causes (hearing loss, middle ear disease, depression, and other comorbidities), and form of treatment. However, the majority (~80%) of afflicted persons have some degree of hearing loss (>2000 Hz) and their tinnitus is a byproduct of hearing loss [3,7,8,9,10]. Most (~80%) people with tinnitus report that their tinnitus pitch is above 2000 Hz, and at least half of them report that their tinnitus is tonal [11,12]. For simplicity, here, we only consider tonal tinnitus in persons with hearing loss (tinnitus henceforth). The objective of this review is to provide a comprehensive background of hearing-loss-related tinnitus, offer a theoretical framework for a multisensory approach to tinnitus remediation, and end with potential studies to test the theoretical framework.

### 1.1. Background

One prevailing theory advocates that tinnitus is caused by deafferentation, or the loss of bottom-up input, from the cochlea to the inferior colliculus and tonotopic region of Heschl’s gyrus [3,10,13,14,15,16,17,18,19]. This form of sensory loss, i.e., deafferentation, leads to an imbalance of inhibitory and excitatory mechanisms, causing hallucinations (e.g., tinnitus) [20]. A consequence of this deafferentation is a cascade of maladaptive neural reorganizations, whereby neurons in the hearing-loss region (>2000 Hz) of Heschl’s gyrus increase their spontaneous or synchronous activity (hyperactivity), partly due to the loss of thalamo-cortical regulatory inhibition of the affected region [13,21,22,23,24,25].

Due to this maladaptive neuroplasticity, previous studies have shown that neurons at the edge frequencies of the hearing-loss region receive input from their normal-hearing neighbors, causing them to respond to these edge frequencies. In other words, neuroplastic adaptation occurs, whereby the neurons of the deafferented region start to encode the frequencies belonging to the healthy regions. Hence, the edge frequencies of the normal-hearing region become overrepresented in tinnitus [26,27]. Furthermore, the consequence of such maladaptive reorganization is that individuals gain enhanced frequency discrimination abilities for edge frequencies [24]. This effect is manifested in enhanced N1 auditory evoked potential (AEP) for edge frequency tones [28]. However, other studies [24,29,30] have shown that enhanced N1 amplitude in tinnitus listeners is also observed for frequencies within the normal-hearing range (e.g., 500 Hz, 1000 Hz), suggesting a broader neural maladaptation due to deafferentation. Additionally, Jacobson et al. [30] reported that the N1 is augmented to attended versus ignored 1000 Hz tones in tinnitus listeners, but not in normal hearing listeners, indicating that selective attention is a factor that should be considered in tinnitus research.

The N1 AEP is a prime auditory evoked potential (AEP) often utilized to study frequency encoding in the auditory cortex, as several studies have shown that tonotopic organization (frequency encoding) of the auditory cortex is reflected in N1 morphology [31,32].

A pertinent finding from a case study [33] further validated the utility of the N1 as an index of tinnitus hyperactivity. This study measured tinnitus severity and N1 amplitude at regular intervals in a young soldier that had experienced sound trauma due to gunfire. As the soldier’s tinnitus lessened in severity over 256 days of follow-up, so did the N1 amplitude (smaller N1s to 1000 Hz pure tone), signifying that tinnitus severity can be tracked by changes in N1 amplitude. However, despite a trend showing the augmentation of the N1 AEP in tinnitus patients, other studies have failed to detect reliable differences in N1 amplitude between persons with and without tinnitus [34,35]. Sereda et al. [35] showed that, while the N1 auditory evoked field (AEF, magnetic counterpart of the N1 AEP) was reduced in amplitude for the tinnitus dominant pitch frequency relative to edge and normal-hearing frequencies, tinnitus patients did not exhibit significant N1 AEF differences relative to the control groups (normal-hearing persons and persons with hearing loss without tinnitus). Taken together, the current neurophysiological knowledge on tinnitus, while exhibiting conflicting accounts, shows a trend suggesting that the N1 AEP to edge and normal hearing frequencies (e.g., 1000 Hz) can serve as gauges of tinnitus sensation and, eventually, recovery. That is, smaller N1s for tones in the normal hearing (lower) frequencies may indicate reduced hyperactivity. Herein, we adopt this view. Note, the main sources that give rise to the N1 AEP lie in and surrounding Heschl’s gyrus (belt and parabelt; [36]), but we cannot rule out contributions from other regions of the auditory cortex. 

One approach to tinnitus treatment is to increase inhibitory input to the affected neurons so that hyperactivity associated with tinnitus can be reduced. For example, Brozoski et al. [37] (also see [38]) reduced hyperactivity along the auditory pathways of rats with neurophysiological evidence of tinnitus by administering a neural inhibitory substance (Vigabatrin, a GABA transaminase inhibitor also used in epilepsy). Increasing inhibitory input has also been considered in humans with tinnitus. Okamoto et al. [39] (see also [40]) used notched music training therapy—music was notched around the pitch of subject’s tinnitus—to induce the lateral inhibition to deafferented neurons by their neighboring neurons. Lateral inhibition is a phenomenon that occurs when neurons coding for certain features are inhibited due to the excitation of neighboring neurons. They showed that a decrease in tinnitus sensation was observed at 6 months, and to a greater extent at 12 months, after training. This effect was reflected in smaller N1 AEFs to a 500 Hz tone (within the normal-hearing range).

Recent studies also suggest the impact of multisensory stimulation, promoting inhibitory signals, on tinnitus remediation. Marks et al. [41] used somatosensory–auditory coupled stimulation (repeated for 20 min), separated by a set time interval known to induce inhibition, to induce a long-term reduction in tinnitus-related hyperactivity in the dorsal cochlear nucleus (DCN). Bimodal, but not unimodal, stimulation resulted in the inhibition of DCN activity and reduced tinnitus severity in both animals and humans after 25 days of daily (20 min/day) stimulation. Finally, Spiegel et al. [42] conducted a study, in which participants with unilateral tinnitus underwent daily multisensory tasks using auditory, visual, and tactile stimuli. In one group (integration group), the three types of stimuli were presented on the tinnitus side, while in the second group (attention diversion group), the three stimuli were presented on the opposite side. Subjects gave a response during each trial. Both groups showed slight but significant mitigation of tinnitus after 20 days (20 min/day) of training, with no significant differences observed between the two groups.

The abovementioned multisensory studies, combined with our own work, motivated us to develop an audiovisual theoretical framework for tinnitus remediation. Our goal is to establish that a reduction in the hyperactivity of deafferented neurons in the auditory cortex can be achieved via daily audiovisual training that specifically targets deafferented neural regions, coding for high frequencies. Our approach extends beyond earlier efforts aimed merely at increasing inhibition via multisensory stimulation. We strive to also repurpose (e.g., “restore”) the function (reverse maladaptation) of deafferented neurons by cross-modally targeting frequency representations in the hearing-loss range with alternating cycles of inhibition and excitation, which we term modulation, to induce a lasting outcome of tinnitus relief.

To summarize, hearing-loss-induced tinnitus is likely a maladaptive byproduct of deafferentation, resulting in the hyperactivity of neurons along the auditory pathway including the auditory cortex. This hyperactivity may be remedied by inhibiting the deafferented neurons. A possible neurophysiological indicator of tinnitus severity is the N1 AEP. There remain gaps in knowledge which include the use of noninvasive approaches to multisensory remediation of tinnitus and the potential use of other biomarkers to assess tinnitus severity and remediation. We propose to use audiovisual training as a way to induce inhibition and modulation (targeted inhibition and excitation), to desynchronize the deafferented neurons, and to achieve a sustained mitigation of tinnitus.

We continue by situating the problem in an audiovisual mechanistic framework and follow up with the theoretical framework, outlining how enhancing reliance on visual cues in daily communications may lead to the reorganization of deafferented neurons in the auditory cortex and ultimately reduce tinnitus sensation. We also suggest the use of alpha band (8–12 Hz) oscillatory activity as an alternative or a complementary biomarker to the well-established N1 AEP. We end by offering one experimental design guided by the theoretical framework that can potentially be used to achieve the neuroplastic reversal of neural hyperactivity and a reduction in tinnitus severity.

### 1.2. The Primary Visual Influence on the Auditory Cortex Is Inhibitory

**Figure 1:**
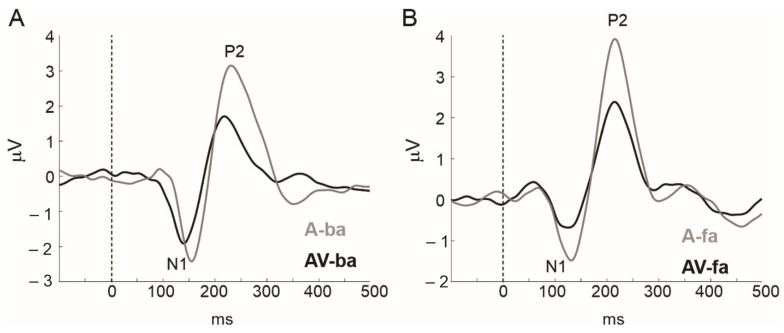
(**A**) Auditory evoked potentials (mean across channels FCz/Cz; *n* = 19) for auditory-only (A, grey) and audiovisual (AV, black) CVs (/ba/ in (**A**) and /fa/ in (**B**)). Data from [50].

Studies on audiovisual integration have consistently demonstrated that when auditory stimuli are paired with visual stimuli, the auditory response is inhibited. Most of these studies, which used speech stimuli and electroencephalography (EEG), consistently demonstrated the suppression of the P1, N1, and /or the P2 AEPs during audiovisual versus auditory-only stimulus presentations [43,44,45,46,47,48]. This suppressive effect has been confirmed by work from our labs [49,50,51]. Reanalysis of data from Shahin et al. [50] revealed that the N1 AEP of the consonant–vowel (CV) /fa/ and P2 AEPs of /ba/ and /fa/ were suppressed when they were combined with videos of a speaker uttering them relative to AEPs of auditory-only tokens (*p* < 0.05; Figure 1). One notable finding showed that this suppressive effect was related to the ability of the visual input to predict the timing of the acoustic stimulus, regardless of whether the visual stimulus contained contextual information relevant to the auditory stimulus (non-speech stimuli) [52]. In other words, this cross-modal inhibitory effect is strongest when the temporal relation is strongest between the two modalities. Indeed, work from our labs showed that this cross-modal inhibitory effect is strongest when temporally misaligned audiovisual stimuli are perceived as in-sync vs. out-of-sync [49,51]. Furthermore, individuals adapt to repeated exposure to asynchronous audiovisual stimuli, such that perceivers tolerate longer windows of asynchrony (perceive in-sync) with more exposure to misaligned audiovisual stimuli [53,54,55]. Given that the cross-modal influence can be gauged by changes to the N1 AEP—a viable neural marker of tinnitus—the role for the audiovisual remediation of tinnitus can be assessed using the N1 AEP. For example, Zeng et al. [56] showed that reduced N1 AEP is directly correlated with reduced tinnitus using low-rate electric stimulation. Taken together, we may posit that adaptation to asynchronous audiovisual stimulation inhibits AEPs to a specific sound, e.g., specific frequency, and thus can be used to suppress tinnitus if the sound’s frequency matches the tinnitus pitch. This should be validated with a reduction in N1 to edge frequencies.

Findings from speech and non-speech stimuli show that this suppressive effect relates to the ability of the visual input to predict the occurrence of an acoustic stimulus [45,49,51,52]. This visual-to-auditory predictive ability, however, is temporally flexible, tolerating about ~200–250 ms of asynchrony between the two modalities [49,51]. In short, this cross-modal inhibitory effect is strongest when vision temporally precedes audition, giving the ability to predict incoming auditory stimuli. Because vision often leads to audition in spoken language [57,58], this suppressive effect, indexed by reduced N1-P2 AEPs, is a powerful tool with which to gauge visual predictive ability on ensuing speech.

**Figure 2:**
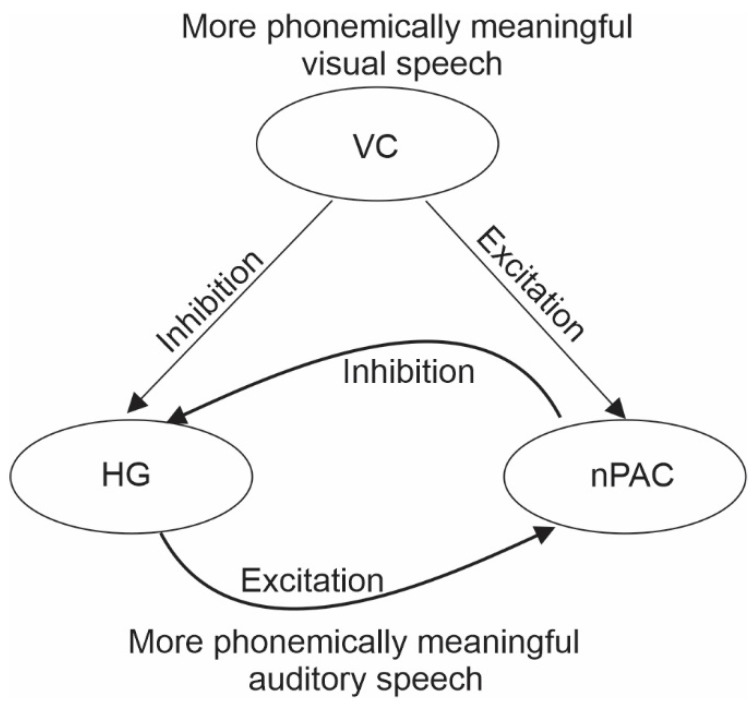
The Dynamic Reweighting Model. Schematic showing how more meaningful visual and auditory information lead to suppression of Heschl’s gyrus (HG).

The mechanisms that underlie this cross-modal suppressive effect is an active debate. Besle et al. [44,48], who based their interpretation on the suppression of AEPs for AV vs. auditory-only stimulus designs, hypothesized that the effect can be ascribed to a reduced auditory engagement due to the predictive processing of some auditory features by the visual modality. In support of this interpretation, Pilling [46] proposed that the suppressive effect occurs following successful audiovisual integration via top-down inhibition of the auditory cortex from multi-sensory networks. Our explanation builds on these theories. In our Dynamic Reweighting Model [49] (summarized in Figure 2), we proposed that the visual-to-auditory inhibitory effect occurs when meaningful visual information shifts processing from Heshl’s gyrus to the non-primary auditory cortex, in turn inhibiting Heschl’s gyrus, either directly or via feedback loops from the non-primary auditory cortex. Hence, the suppressed AEPs.

### 1.3. The Secondary Visual Influence on Auditory Representations Is Modulatory

**Figure 3:**
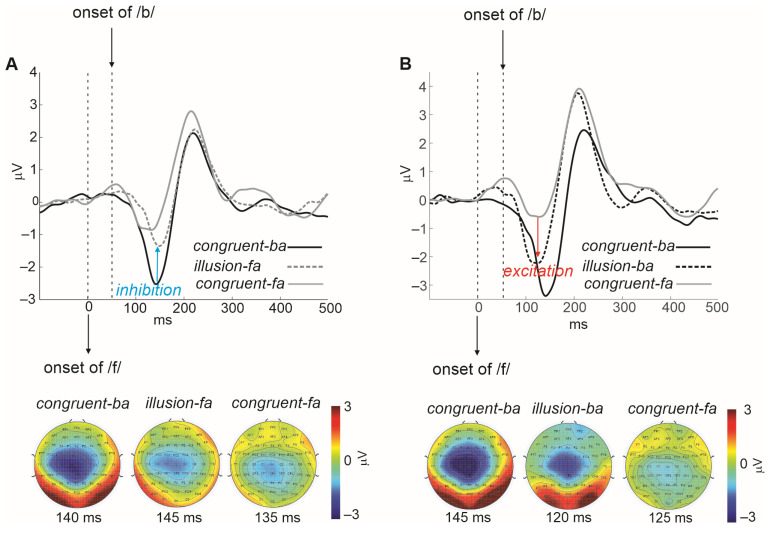
(**A**) AEP waveforms of illusion-fa (/ba/ heard as ‘fa’) vs. of audiovisual congruent-ba and congruent-fa stimuli (*n* = 17). (**B**) AEP waveforms of illusion-ba (/fa/ heard as ‘ba’) vs. of congruent-ba and congruent-fa stimuli (*n* = 9). AEPs are time-locked to /fa/ onset, which occurs 50 ms earlier than the voicing (/ba/). Data from [50] reproduced under the terms of the Commons Attribution 4.0 International License (CC-BY).

We recently demonstrated that in spoken language processing, the visual effect on the auditory cortex is not only inhibitory but also modulatory [50,59]. The inhibitory effect discussed in the previous section is believed to be non-contextually specific. By ‘modulatory’, we imply a secondary contextually specific form of cross-modal inhibition and excitation. Specifically, following the broad (non-specific) cross-modal inhibition of the auditory cortex (as in Figure 1), the visual system proceeds to further modulate (inhibit/excite) speech features in auditory cortex neurons to shift percepts toward those conveyed by the visual system (see Figure 3). 

In Shahin et al. [50], we presented individuals with audios of the CVs /ba/ and /fa/, combined with congruent and incongruent videos of the speaker uttering the same syllables (Figure 2). We also presented subjects with the same stimuli without visual input (auditory-only). Listeners performed closed-set syllable identification (‘ba’ or ‘fa’). The experiment was designed to visually alter auditory perception. The CV /fa/ is heard as ‘ba’ when the initial fricative /f/ is removed because the voicing portions of both syllables have similar formant trajectories. When visual /ba/ is combined with audio /fa/, listeners often report hearing ‘ba’. For this to happen, visual networks need to inhibit the neural representation of the initial fricative, /f/, in the auditory cortex, which has a wide frequency band (e.g., 100–10,000 Hz, including frequencies typical of the HL region). When video /fa/ is combined with audio /ba/, listeners often report hearing ‘fa.’ In this this case, visual networks need to *excite* the /f/ auditory cortex representations, activating neurons that code for a wide band of frequencies. Indeed, the pattern of neural activity for the illusory percept mirrors that seen for auditory-only /ba/ or /fa/. In general, responses for audio /ba/ are smaller than those for audio /fa/. However, hearing the illusory ‘fa’ (/fa/ video, /ba/ audio) evokes a reduced N1 that resembles the N1 to audio /fa/. Similarly, hearing the illusory ‘ba’ (/ba/ video, /fa/ audio) evokes an enhanced N1 that resembles the N1 to audio /ba/. In short, using the same data, we show that visual influence on the auditory cortex is not only inhibitory (Figure 1), but also modulatory (Figure 3); where the N1 is altered to resemble that of the visually conveyed auditory percept.

In summary, previous accounts, including our own, revealed that when auditory stimuli are accompanied by visual stimuli, AEPs become smaller, emphasizing that the initial visual influence on the auditory cortex is inhibitory. In addition to this initial effect, visual context instigates a shift in the N1 amplitude to reflect the N1 of the visually, as opposed to the acoustically, conveyed phonemes, emphasizing that visual influence on audition is also modulatory. Our current theoretical framework is motivated by previous attempts at tinnitus remediation, which are summarized in Table 1. However, these accounts only probed the inhibitory aspect of tinnitus remediation. By also exploring the effect of cross-modal modulation (targeted inhibitory and excitatory stimulation) on specific sound representations, we endeavor to reverse the neuroplastic maladaptation of tinnitus (“restore neural function”) and cause a lasting tinnitus relief.

## 2. Theoretical Framework

**Figure 4:**
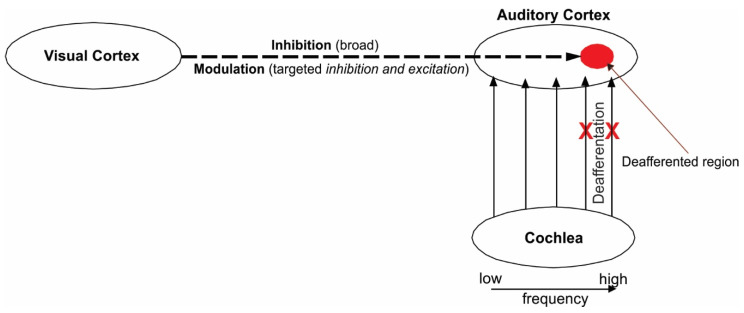
Theoretical framework. Neural model depicting how visual input to auditory cortex can counterbalance the loss of bottom-up input to auditory cortex from the cochlea. By cross-modally inhibiting/modulating the deafferented region of auditory cortex, activity of hyperactive neurons not only become inhibited, but also function of these neurons may be restored if they begin to encode meaningful context provided by visual networks.

Our theoretical framework for tinnitus remediation stems from the idea that alternating the suppression and enhancement of activity within the deafferented region of the auditory cortex can be achieved via audiovisual training, such that (Figure 4): (1) if a loss of input from the cochlea to the tonotopic region of the auditory cortex leads to enhanced synchrony and hyperactivity of the deafferented neurons, (2) The inhibition of the activity of the deafferented auditory neurons reduces tinnitus sensation, (3) Visual networks modulate representations in the auditory cortex; then, the loss of bottom-up input from the cochlea, and hyperactivity in the auditory cortex, can be compensated via enhanced cross-modal modulation of the deafferented region (targeting high-frequency neurons) following a period of audiovisual training. That is, the affected neurons can relearn to encode the spectral information via visual input (e.g., audiovisual combination of /ba/ and /fa/ discussed above), as if there were acoustic input. This “restoration” of function via cross-modal modulation should reduce synchrony of deafferented neurons more so than broad inhibitory mechanisms alone, which was tried previously, leading to a lasting reduction in tinnitus severity.

## 3. Audiovisual Training as a Means to Remedy Tinnitus

Given our tentative theoretical framework, we propose that sustained audiovisual training, whereby individuals are exposed to visual context that directly alters the perception of high-frequency sounds, e.g., fricatives, can be a useful intervention for tinnitus. We outline a hypothetical study motivated by the abovementioned theoretical framework below.

### 3.1. Audiovisual Filling-In of Notched Speech

One approach is to build on Okamoto et al. [39]. Persons with tinnitus can regularly (daily) watch narrations of novels, whereby the talker’s mouth movements are closely observed, with the speech stream frequency notched (frequency removed) around the person’s own tinnitus frequency. To gauge progress associated with training, tinnitus severity profile and EEG (specifically the N1 AEP) can be obtained at several intervals during and after training.

First, from an auditory-only perspective, this will allow lateral inhibition [39] to regularly take place, causing deafferented neural populations to desynchronize, leading to a reduction in tinnitus over time. Second, visual input will trigger a secondary inhibitory input, on top of the lateral inhibitory input, strengthening the overall inhibitory mechanisms targeting deafferented neurons. Third, the inclusion of visual cues will increase cross-modal phonetic encoding, activating (exciting), and deactivating (inhibiting) the deafferented auditory region—on top of the broad inhibitory effect. We know from previous work that linguistic and visual contexts enhance phonemic restoration, also known as the continuity illusion or illusory filling-in. The continuity illusion or phonemic restoration is an illusion whereby speech with a noise-replaced segment is perceived as being continuous through the noise period [60]. Phonemic restoration is facilitated by perceptual, linguistic, and cognitive factors [61,62,63]. Several studies have also shown that phonemic restoration is mediated by visual context [50,59,62,64,65]. In Shahin et al. [50], we showed that the N1 AEP amplitude shifts to exhibit the amplitude of the visually conveyed phoneme, despite an incongruent auditory phoneme. Our work confirmed that phonemic encoding is altered by the visual modality. This underlies cross-modal phonemic restoration.

**Figure 5:**
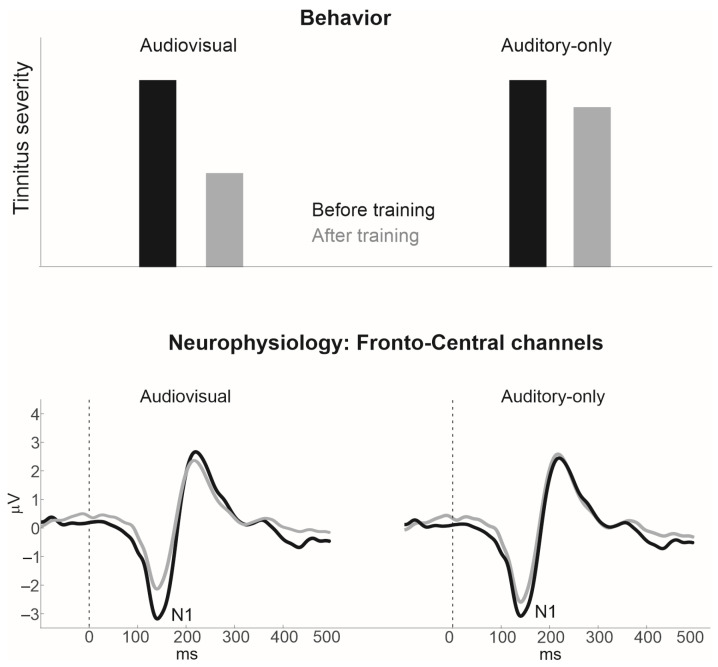
Hypothetical results. **Top panel:** tinnitus severity as a function of audiovisual and auditory-only training. **Bottom panel**: amplitude of the N1 auditory evoked potential as a function audiovisual and auditory-only training.

In short, the visual-to-auditory suppressive effect combined with the visual-to-auditory modulatory effect will not only reduce hypersynchrony of the deafferented neural population but will also repurpose the function of these neurons to encode phonemic information via the visual system, further reducing hypersynchrony. The end result of such a combination is the reduction in tinnitus severity. As in Okamoto et al. [39] and Pantev et al. [33], this neurophysiological and behavioral transformation can be gauged by the corresponding change in the N1 auditory evoked response. That is, one should expect a systematic reduction in N1 amplitude as a function of training, as we hypothetically demonstrate in Figure 5.

### 3.2. Potential Limitations

In terms of limitations, our proposal’s success hinges on the assumption that connectivity between the auditory and visual modalities not only exists but is as strong in tinnitus persons as it is for normal-hearing non-tinnitus persons. Cross-modal inhibition and modulation can only be effective when inter-modal connectivity is strong. Preliminary data (not shown) from our lab do not support the existence of an equal visual-to-auditory effect in tinnitus persons. We examined AEPs to audiovisual and auditory-only stimuli in one person with tinnitus (age 29) with a hearing loss notch at about 5000 Hz and an age matched normal hearing non-tinnitus person. The two individuals passively listened to 2000 Hz tones and speech sounds while watching a silent anime movie designed to convey a story without sound or subtitles. In order for the individuals to understand the story, they must engage their linguistic networks. Consequently, this will lead to the inhibition of Heschl’s gyrus as we outline earlier, including in Figure 2, resulting in smaller N1. AEPs of the tinnitus male subject showed a lack of visual-to-auditory inhibitory N1 effect compared to age-matched non-tinnitus (normal-hearing) male. However, before one can address such a potential limitation, data need to be collected from more individuals.

### 3.3. Alternate Approaches, Moving beyond the N1 AEP

In terms of alternate approaches, one of the most studied approaches in EEG is via assessing the behavior of alpha band activity (8–14 Hz). Enhanced alpha activity is known to index neural disengagement (inhibition) of task irrelevant neural networks, while suppressed alpha activity indicates the engagement (excitation) of task-relevant neural networks [63,66,67,68,69,70]. Pertinently, the link of alpha to tinnitus severity was evidenced in one case study. In one person with unilateral cochlear implant who experienced tinnitus, Zeng et al. [56] demonstrated that patient tinnitus was significantly suppressed by a low-rate (<100 Hz) electric stimulation to the apical part of the cochlea. The stimulation resulted in reduced N1 AEP, accompanied by enhanced alpha power originating from the auditory cortex. Both the reduced N1 and enhanced alpha are indicators of reduced hyperactivity.

## 4. Conclusions

While previous accounts on tinnitus have made a substantial behavioral and neurophysiological advance in building knowledge regarding the causes and treatment of tinnitus, there remain gaps in knowledge that warrant further investigations. In our view, one of these gaps is the link between audiovisual mechanisms and tinnitus evaluation and treatment. We propose a theoretical framework regarding audiovisual training as a means of tinnitus remediation. Our framework is grounded in how natural audiovisual processing can be utilized to alter the behavior of hyperactive neurons giving rise to tinnitus along the auditory pathways.

## Figures and Tables

**Table 1:** Summary of 6 studies that motivated the current theoretical framework.

Reference	Research Method	Main Findings
[21]	Vigabatrin (a neural inhibitory substance) was administered to rats exhibiting neurophysiological evidence of tinnitus to reduce hyperactivity in the auditory pathway.	Brainstem activity, which is increased in rats with neurophysiological evidence of tinnitus, was lowered by the application of Vigabatrin.
[41]	(1) Bimodal somatosensory–auditory coupled stimulation, separated by a time interval that is known to induce long-term depression, were administered to guinea pigs for 20 min a day for 25 days. (2) The same stimulation treatment was applied to 20 human subjects with tinnitus for 28 days. In this case, the treatment involved both bimodal and unimodal conditions.	(1) Behavioral and physiological evidence of tinnitus were reduced in the guinea pigs. (2) Tinnitus loudness and intrusiveness were reduced in those exposed to the bimodal treatment. There were no significant benefits observed using unimodal auditory stimulation.
[39]	Music listening therapy, whereby the music was notched around the tinnitus pitch of each individual. Tinnitus sensation was measured at 6 and 12 months after the start of music therapy.	A decrease in tinnitus sensation was observed at 6 months, and an evenlarger reduction was seen at 12-month. Accompanying the decreased tinnitus sensation was a decrease in N1 auditory evoked field to a 500 Hz tone.
[40]	(1) Tailor-made notched music training (TMNMT), whereby the music energy spectrum was notched around the tinnitus pitch of each individual, was administered to a population with unilateral tinnitus ≤8000 Hz (*n* = 39, split between treatment and control groups) at a rate of 1–2 h per day for 12 months. Tinnitus sensation and auditory evoked cortical activity were recorded every 6 months. (2) TMNMT was administered to groups with ≤8000 Hz (*n* = 10) or >8000 (*n* = 10) Hz unilateral tinnitus frequencies at 6 h per day for 5 days.	(1) Tinnitus loudness and annoyance levels were reduced after training. Tinnitus-related auditory evoked fields were also significantly reduced. There was no change from baseline in the control/placebo group. (2) Tinnitus loudness and distress, as well as auditory evoked activity, were significantly reduced in the ≤8000 Hz tinnitus group. No significant changes were observed in the >8000 Hz tinnitus group.
[42]	Multisensory tasks (auditory, visual, and tactile) were administered at 20 min daily for 20 days to a population with unilateral tinnitus (*n* = 18). In one group (integration group), the stimuli were administered on the tinnitus-affected side, while in another group (attention diversion group), the stimuli were administered on the opposite side. Tinnitus levels were reported before and after the training.	After 20 days, both groups showed a significant reduction in tinnitus. There was no significant difference between the two groups.
[56]	A low-rate (<100 Hz) electrical stimulation was applied to the apical portion of the cochlea of a person with a unilateral cochlear implant. Tinnitus levels were measured, as well as N1 auditory evoked potential and alpha power.	During stimulation, tinnitus sensation was reduced, N1 auditory evoked potential was reduced, and alpha power generated from the auditory cortex was enhanced.
